# Toward Verification of DAG-Based Distributed Ledger Technologies through Discrete-Event Simulation

**DOI:** 10.3390/s24051583

**Published:** 2024-02-29

**Authors:** Misbah Khan, Frank den Hartog, Jiankun Hu

**Affiliations:** Canbbera Campus, University of New South Wales Canberra at the Australian Defence Force Academy, Campbell, ACT 2612, Australia; misbah.khan@unsw.edu.au (M.K.); frank.den.hartog@unsw.edu.au (F.d.H.)

**Keywords:** blockchain, coordinator, decentralization, directed acyclic graph, distributed ledger technology, IOTA, micro-transactions, scalability, Tangle

## Abstract

As the potential of directed acyclic graph (DAG)-based distributed ledgers in IoT systems unfolds, a need arises to understand their intricate dynamics in real-world scenarios. It is well known that discrete event simulations can provide high-fidelity evaluations of protocols. However, there is a lack of public discrete event simulators capable of assessing DAG-based distributed ledgers. In this paper, a discrete-event-based distributed ledger simulator is introduced, with which we investigate a custom Python-based implementation of IOTA’s Tangle DAG protocol. The study reveals the dynamics of Tangle (particularly Poisson processes in transaction dynamics), the efficiency and intricacies of the random walk in Tangle, and the quantitative assessment of node convergence. Furthermore, the research underscores the significance of weight updates without depth limitations and provides insights into the role, challenges, and implications of the coordinator/validator in DAG architectures. The results are striking, and although the findings are reported only for Tangle, they demonstrate the need for adaptable and versatile discrete event simulators for DAG architectures and tip selection methodologies in general.

## 1. Introduction

The Internet of Things (IoT) is ushering in a new era characterized by the interconnectivity of billions of devices. With this vast network comes the critical challenge of ensuring data integrity and decentralization. While blockchain technology has been lauded for its unparalleled characteristics of integrity, decentralization, and auditability, its linear data structure has shown potential limitations in meeting the high transactional demands of IoT systems.

DAG-based distributed ledgers represent a significant evolution from traditional blockchain technology. Unlike blockchains, which operate on a linear sequence of blocks starting from a genesis block, DAGs expand in a non-linear, multi-dimensional manner. This structure allows multiple blocks to be added to the network simultaneously, leveraging the DAG data structure, where each node can represent a block or a single transaction, depending on the specific architecture of the blockchain in question. This capability for parallel transaction processing markedly enhances the scalability and makes DAG-based systems particularly well suited for high-volume transaction environments, such as those found in the Internet of Things.

DAG-based ledgers like Phantom, IOTA [1], Nano [2], and Hashgraph [3] have showcased the vast potential of this technology by facilitating faster transaction speeds and lower operational costs, thereby addressing some of the scalability and efficiency challenges faced by conventional blockchains. However, it is crucial to acknowledge the limitations and challenges that DAG-based systems encounter, including network security, consensus mechanism complexities, and the trade-offs between decentralization and scalability. Understanding these aspects is essential in utilizing the full potential of DAG-based distributed ledgers to create more efficient, scalable, and secure decentralized systems.

This paper presents a discrete-event-based evaluation of the Tangle protocol, following its description in [1] as closely as possible. We focus on Tangle’s initial version to grasp its core principles, setting a solid foundation before exploring the complexities of its recent (but significantly less well described) evolution toward a validator committee [4]. This approach ensures a thorough understanding of the fundamental design before delving into advanced developments. The discrete event simulator is developed such that it is both flexible and extensible, to support future research across other DAG-based ledger systems. For instance, the simulator in its current form incorporates Tangle’s random walk strategy; however, its architecture is designed to be adaptable, allowing for the straightforward integration of other methodologies, such as Virtual Block [5]. Unlike the asynchronous, continuous-time model used in the DAGsim multi-agent simulation framework for DAG-based cryptocurrencies [6], our simulator operates on the principle of discrete events, providing a distinct and precise method of evaluating system behavior and performance. In contrast to another work [7] that introduces an agent-based simulator focusing primarily on evaluating the performance of the Tangle protocol under diverse network conditions and attack scenarios, our proposed simulator offers a more comprehensive approach. Our work not only features an agent-based model with individual agents representing network nodes, each exhibiting distinct behaviors like transaction issuance, validation, and decision-making based on predefined rules, but also extends to model-based simulations. In the latter, we leverage a quantitative model to depict the continuous dynamics of the DAG-based blockchain. These equations capture critical aspects such as the accumulative weight, Poisson distribution, node convergence over time, and network propagation dynamics. The only known discrete event simulator in the literature designed for DAG-based distributed ledgers is DAG-Sword [8], which builds upon a previous Bitcoin mining simulator [9] to verify miner block rewards. While DAG-Sword primarily focuses on DAG-based consensus protocols and the impact of malicious miners on the transaction processing throughput in protocols like Phantom and GhostDAG, it leans more toward transaction fees and a mempool data structure due to the linear behavior of its underlying Bitcoin mining simulator. In contrast, our work presents a discrete event simulator that offers a more comprehensive perspective on DAG-based ledgers. Our study encompasses discrete event evaluation, meticulously simulating discrete events within the DAG-based blockchain and encompassing timestamped actions like transaction creation, transaction propagation, transaction validation (by new tips), transaction approval (by milestones), tip selection, milestone creation, and double spending. This meticulous approach ensures that these events are processed in precise chronological order. Our multifaceted research methodology provides a robust and comprehensive foundation for the in-depth analysis and potential enhancement of DAG-based distributed ledger systems.

Our contribution is twofold: (1) providing a comprehensive understanding of Tangle and (2) establishing a benchmark for the evaluation and improvement other DAG-based distributed ledger technologies.

The remainder of the paper is organized as follows. Section 2 reviews related work. Section 3 elaborates on our methodology and the specifics of our discrete event distributed ledger simulator. Section 4 presents the results coupled with a comprehensive analysis. Section 5 presents a discussion and outlines potential future work. Section 6 provides the concluding remarks and our contributions to the field of DAG-based distributed ledger systems.

## 2. Related Work

Recent research has seen the development of various simulators, like Blocksim [10,11] and Blockperf [12], along with subsequent enhancements, such as those to Blocksim [13], providing valuable tools for the in-depth investigation of blockchain solutions. However, when it comes to DAG-based blockchain systems, such efforts are notably scarce in the literature, highlighting a gap in research and development for these types of distributed ledgers.

The article [6] presents a simulation framework for the study of DAG-based cryptocurrencies, specifically focusing on IOTA. This framework models how transactions occur and are accepted in such systems by simulating the behavior of both honest and semi-honest actors. The study finds that agents (or nodes) in the network with low latency and high connectivity have a better chance of having their transactions accepted. The framework has been designed with extensibility in mind, allowing for the inclusion of other DAG-based protocols and the potential addition of malicious agents in the future. However, while this is a commendable effort, the approach emphasizes solely thread-based implementation. As outlined in Section 3.1, it is limited to utilizing eight threads due to system constraints. In contrast, the methodology presented in our work encompasses not only thread-based implementation, but also asynchronous tasks. Therefore, if researchers have access to advanced supercomputers like NCI Gadi, our approach can be effortlessly expanded to multi-CPU configurations, enabling the generation of more realistic and comprehensive results.

Another study [14] investigates the performance and scalability of IOTA. Using an extended version of the DAGsim simulator [6], the study delves into factors such as the transaction arrival rate, tip selection algorithms, and network delay to provide insights into IOTA’s performance. Further, another DAG-based simulator, TangleSimulator, has been proposed [15], focusing on the stability of tip counts against various tip selection methods. An additional study [16], builds upon the TangleSimulator, offering enhanced configuration options and a larger number of transactions. This work illustrates and examines the fundamental components of Tangle. However, similar to the study in [6], it also demonstrates smaller configuration parameters. Another study [7] introduces an agent-based simulator focusing on the performance of the Tangle 2.0 protocol under various network environments and attack scenarios. This study provides a comprehensive understanding of Tangle, yet it focuses exclusively on IOTA. Furthermore, MAIOTASim [17] also proposes a multi-agent IOTA simulator, providing the security verification of consensus under double spending attack scenarios. However, this work also only focuses on the IOTA protocol, unlike our proposed simulator, designed with generic components for overall DAG-based distributed ledgers.

A notable work by [18,19] provides a comprehensive empirical analysis of IOTA’s Tangle using real transaction data officially released by the IOTA Foundation. Their study showcases Tangle’s topological features and observed performance, contrasting it with the prevailing literature’s conclusions. Specifically, they shed light on the actual transaction confirmation time and the topological characteristics of real IOTA tangles, which differs from commonly held beliefs about IOTA’s efficiency compared to traditional blockchains. Guo et al.’s [18] analysis illuminates certain latency issues with Tangle, highlighting that the actual transaction confirmation time may not be as efficient as once assumed. This work is instrumental in presenting an empirical perspective on Tangle, emphasizing the importance of real-world data in evaluating decentralized platforms. However, while their work offers profound insights into Tangle’s empirical analysis, our study delves deeper into the practicality of the IOTA protocol. Our research focuses on the finest discrete events occurring in Tangle, providing a more holistic view of the IOTA protocol’s behavior in high-fidelity simulation environments, which can serve as a robust baseline for future investigations into DAG-based distributed ledgers.

In summary, the research landscape on IOTA’s Tangle and DAG-based distributed ledgers has seen a spectrum of investigations, ranging from empirical analyses to simulation-based studies. While these studies have provided invaluable insights into the workings and challenges of the IOTA protocol, there remains substantial scope for enhanced methodologies that transcend traditional approaches. This research endeavors to fill this gap, offering a novel perspective and approach that further elevates the understanding of the Tangle protocol. A comparison of the DAG-based simulators is presented in Table 1.

## 3. Simulator Implementation with Tangle Protocol

The following section delineates the step-by-step process followed to develop the simulator curated with the Tangle protocol, as presented in its white paper [1]. This hands-on approach aimed to replicate the theoretical underpinnings in a real-world environment to ensure the practical applicability and operability of Tangle.

### 3.1. Environmental Setup

The implementation of the simulator and Tangle protocol was constructed using Python, chosen for its ease of use, clear syntax, and comprehensive libraries that align with the specific needs of distributed ledger technology. By leveraging Python’s asyncio [20] and Threading libraries [21], asynchronous network communication between nodes was effectively handled, ensuring smooth and concurrent node connections.

Python’s (3.10) *hashlib* and *RSA* [22] libraries, renowned for their robust cryptographic hash functions, were utilized to create unique and secure transaction identifiers. For management and operating on the DAG structure, data handling libraries like pandas and *NumPy* [23] were employed, simplifying the intricate tasks of data manipulation and processing.

For the visual analysis and debugging of the DAG, *NetworkX* [24], a Python package for the creation, manipulation, and study of the structure, dynamics, and functions of complex networks, was used. In conjunction, *matplotlib* [25] and *Graphviz* [26] provided a flexible method for the visualization of the data, offering insights into the state of the network at any point and aiding in the debugging and optimization processes.

### 3.2. Tangle’s Core Structure

**Figure 1:**
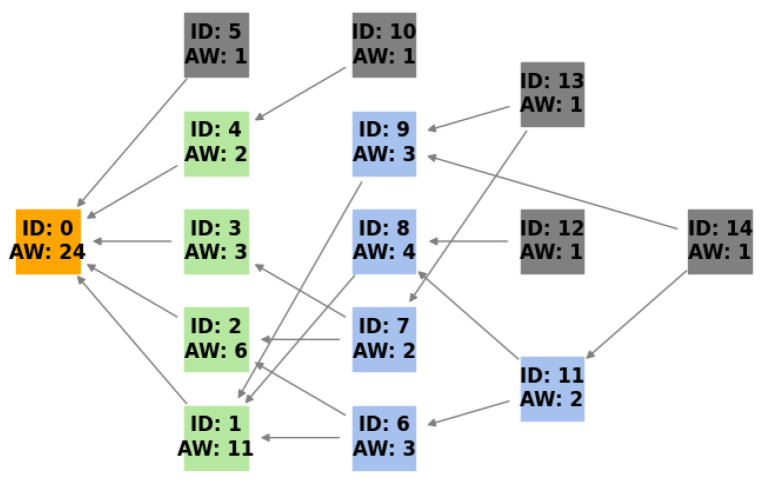
A visual representation of Tangle’s directed acyclic graph. Each transaction is denoted by a rectangle containing its unique ID and its accumulative weight (AW). Transactions reference two preceding transactions, as indicated by the connecting edges. Green rectangles represent transactions that have been approved by milestones. Blue rectangles highlight transactions that, while not approved by a milestone, are referenced by other transactions. Lastly, the grey rectangles symbolize tips, which are transactions that have not yet been referenced by any subsequent transaction.

Tangle is the foundational data structure for IOTA. Unlike traditional blockchains that operate in linear chains of blocks, Tangle is built on a DAG data structure. Each vertex in this DAG represents a transaction, as presented in Figure 1. The structure allows multiple transactions to be added simultaneously; there is no need for miners as the transactions are initially confirmed by the new incoming transaction, ensuring zero transaction fees. This setup is vital for the scalability and micro-transactions on the IOTA network, especially pertinent for IoT applications. Implementing Tangle required the creation of a representation of a DAG in the system. Each transaction or vertex in Tangle contained essential data such as its ID, the IDs of the two transactions that it confirmed (previous vertices), and other transaction-related information. To represent Tangle in this study, a model was developed with the following components.

#### 3.2.1. DAG Representation

The DAG configuration of the network was developed utilizing the NetworkX library. Individual graph representations were generated for each node in the network, offering a localized view of Tangle from different nodes’ perspectives.

#### 3.2.2. Transaction Management

The transaction class handled transactions within the network, encapsulating the characteristics and behaviors of individual transactions. Each transaction had unique identifiers, parent references, an associated node, an accumulated weight, and a timestamp. The class ensured the authenticity and integrity of transaction data through signature mechanisms using the RSA algorithm. An important part of the process was checking that each transaction had performed a certain amount of computational work, known as proof of work. Furthermore, every transaction was assigned its own weight (OW) and accumulative weight. Moreover, a confirmation status flag was also initiated to indicate the transaction’s confirmation status, which would later be used in coordinator validation.

#### 3.2.3. Accumulative Weight

In Tangle’s architecture, the accumulative weight is a very important metric. This weight is the sum of the transaction’s OW (which is set to 1 for simplicity) and the OW of all transactions that directly or indirectly reference it. The system adopted a topological sequence for transaction processing by prioritizing parent transactions over their respective descendants.

The accumulative weight of a transaction was dynamically updated during its processing. Let $W_{x}$ be the own weight of transaction *x*, $C_{x}$ be its set of child transactions, and $AW_{x}$ be its accumulative weight. The relationship between these parameters is given by

(1) $$AW_{x}=W_{x}+\sum_{y\in C_{x}}AW_{y}$$

This equation signifies that the accumulative weight of a transaction is the sum of its own weight and the accumulative weights of its child transactions [1] (Section 2). Transactions without children have their AW equal to their OW, while, for those with children, their children’s accumulative weights contribute to the parent’s overall weight. This meticulous approach to computing the accumulative weight ensures that the metric authentically reflects a transaction’s relative importance and influence within Tangle.

#### 3.2.4. Asynchronous Simulation

In the simulation, we mimicked how a node works in the network, especially how it continuously adds transactions following a Poisson distribution. We used an asynchronous model for this, which captured the network’s nature of handling many operations at once.

#### 3.2.5. Coordinator

In Tangle, the coordinator emerges as a central figure, ensuring transactional integrity. In the simulation, the coordinator was initiated with specific parameters, such as the *‘milestones_interval’* and ID, which was configured with all nodes so that they could verify that the milestone was issued by a legitimate coordinator. The coordinator’s primary responsibility was to create the foundational *‘genesis_milestone’*, a unique transaction without predecessors that acts as a root transaction of the DAG. Moreover, the coordinator efficiently broadcast this milestone across the network. The coordinator guaranteed that milestones were consistently generated at regular intervals.

### 3.3. Random Walk

In Tangle, every new transaction was tasked with referencing two previous transactions. This reference protocol was directed by the tip selection algorithm (TSA). The TSA employed the random walk technique to ascertain consensus on transaction confirmations. Given this algorithm, the most recent transactions, labeled as ’tips’, inherently had an augmented likelihood of attaining approval from forthcoming transactions. This implementation aligns with the principles outlined in Section 4 of the Tangle white paper [1]. The random walk serves as the central mechanism for the tip selection process in Tangle. It comprises two primary walks: the unbiased random walk (URW) and the biased random walk (BRW). The choice between URW and BRW relied on the parameter α, with the URW being chosen when α=0; otherwise, the BRW was initiated. This α value determined the degree of bias toward transactions with a higher AW.

The BRW used the Markov Chain Monte Carlo (MCMC) approach, and the transition probability from one transaction to the subsequent one, progressing toward the tips, was given by

(2) $$P_{xy}=\frac{\exp(-\alpha \left(H_{x}-H_{y}\right))}{\sum_{z:z\to x}\exp{\left(-\alpha \left(H_{x}-H_{z}\right)\right)}^{-1}}$$

where

- $P_{xy}$ represents the transition probability;
- $H_{x}$ stands for the cumulative weight of the current transaction;
- $H_{y}$ stands for the cumulative weight of the transaction toward which the random walker intends to move;
- α is a bias parameter that determines the degree to which the random walk is biased toward transactions with a particular cumulative weight.

#### 3.3.1. Randomwalker Configuration

The *‘RandomWalker’* class was responsible for performing the random walk. Its configuration parameters included the following.

- ‘W’: defines the time interval, emphasizing the importance of recent transactions;
- ‘N’: determines the number of walkers to be deployed for the consensus process;
- α: a bias parameter to influence the walker’s decision in favoring transactions with specific cumulative weights;
- ‘node’: identifies the specific node in the network initiating the random walk.

#### 3.3.2. Interval Determination

The system checked how old the DAG was compared to a set time interval. If it was newer, the period was changed. Then, transactions that happened during a specific time (from W to 2W s) were collected and used in the random walk.

#### 3.3.3. Random Walk Execution

1. **Transaction Selection**: A random subset of transactions, sized ‘N’ (random walkers), from the chosen interval, initiated the random walk.
2. **Dispatching Walkers**: Each transaction underwent an independent walk toward the tips. This process was based on asynchronous programming and allowed all tasks (random walkers) to happen at the same time.
3. **Walk Type Determination**: The URW was chosen if *‘alpha_low’* equaled 0, signifying a uniform transaction transition. Otherwise, the BRW was employed, and the transition probability was calculated based on Equation (2).
4. **Tip Selection**: Post-walk, if fewer than two unique tips were reached, additional ones were randomly picked. The tips were subsequently sorted, and the first two were selected.

This implementation presented a robust random walk mechanism for Tangle, based on firm theoretical underpinnings.

### 3.4. Network Formation and Dynamics

In our designed simulator, we developed a network with a versatile framework that integrated functions such as *create_peer*, latency management, transaction spread, solidification, and methods to prevent redundancy. These functionalities were wide-ranging and adaptable, suitable for a variety of DAG-based distributed ledgers. Although this adaptability was a significant attribute, the current configuration was specifically optimized for IOTA’s Tangle network. Our system effectively mirrored the key features of Tangle, including its decentralized structure, ability to scale, and distinctive transaction validation process.

#### 3.4.1. Node Connectivity

The *create_peers* method was responsible for crafting the network’s structure. In this setting, the nodes were not fully connected. Instead, each one interacted with an adjustable probability of its counterparts, providing an optimal combination of efficiency and resilience. However, to guarantee the integrity of the network and provide reference milestones, the coordinator was connected to all nodes (adjustable).

#### 3.4.2. Latency Modeling

Real-world network communication involves unpredictable and varied delays. This behavior was replicated in our framework through the *generate_delay_matrix* method. It assigned probabilistic delay values to potential interactions between nodes, based on a predefined range.

#### 3.4.3. Gossip Transaction Mechanism

Transactions were propagated through a gossip mechanism, as illustrated by the *gossip_transaction* method. A node initiated a transaction, sending it to a subset of its peers. This broadcast strategy, dictated by the *subset_factor*, ensured strategic and controlled propagation.

#### 3.4.4. Latency Consideration

Transaction propagation delays were controlled by the latency values derived from the delay matrix. Each transaction endured a unique delay, based on its source and destination nodes defined in the *delay_matrix*.

#### 3.4.5. Recursion and Network Penetration

The gossip transaction mechanism employed a recursive strategy. When a node received a transaction, it became a sender, propagating the transaction to its peers. This iterative approach guaranteed the transaction’s penetration throughout the network.

#### 3.4.6. Avoidance of Redundancy

To prevent redundant transactions for a single node, we implemented conditional checks that were activated when the node received a transaction. This ensured that nodes did not re-process transactions that they had already encountered.

In summary, this approach, integrating node connectivity, latency modeling, and a gossip-based transaction mechanism, lays a foundation for future research in DAG-based distributed ledgers.

### 3.5. Node Functionality

In DAG-based distributed ledgers, nodes are crucial in creating, validating, and broadcasting transactions. These nodes support the network’s expansion, maintaining the network’s continuity and integrity. Their functions are defined by the details of their algorithmic processes. The system described in this study, although based on the Tangle protocol, also shares similarities with other DAG-based distributed ledgers. In this study, we developed a *node* class encompassing the essential operations of a node, including *create_and_sign_transaction*, *receive_transaction*, *broadcast_transaction*; these functions leverage the *network* class for transaction propagation mechanisms like *create_peers* and *gossip_transaction*, etc. The methods outlined are not only applicable to the Tangle system under study but also extendable to other DAG-based systems.

#### 3.5.1. Transaction Creation and Signing

In our implementation, a node initiates a transaction by generating unique data. This process involved creating a random data string of N bytes, which was then decoded using the ‘latin1’ encoding. The transaction, represented by a ‘Transaction’ object, was identified by its transaction ID (‘txid’) and included references to two-parent transactions, forming the DAG structure. Furthermore, security was enforced through a cryptographic proof of work (PoW), and the node signed the transaction with its private key using RSA and SHA-256 hashing, ensuring data integrity. The transaction was then added to the node’s list of transactions and unconfirmed transactions and was also considered a new ‘tip’ in the network. Moreover, the node performed a check to remove any referenced parent transactions from its list of tips. This action ensured that only new tips were retained in the network. The code implementation effectively captured the intricate process of transaction creation, signing, and management within the network.

#### 3.5.2. Transaction Broadcasting and Reception

Once a transaction was created and signed, the node broadcast it to its peers in the network. This propagation ensured the decentralization and redundancy of transactional data across the network. While broadcasting, a delay might have been imposed to simulate network latency, as established by the network’s *delay_matrix*. When a node received a new transaction, it performed a series of checks to ascertain its validity. These checks included examining whether the received transaction or its parent transactions had been previously seen or were part of the node’s transaction list. Furthermore, if the parent transactions were missing, the node requested them, ensuring a holistic understanding of the transaction’s ancestry in the network. It is worth noting that these processes represented more of an operational or implementation detail rather than a core theoretical concept, as presented in the Tangle white paper. This approach of retrieving missing pieces for holistic data understanding is also seen in other peer-to-peer systems, showcasing its utility and pragmatic nature [27].

#### 3.5.3. Milestone Receipt and Validation

Nodes in the implemented Tangle occasionally received milestones (critical markers signifying the consensus state of the network). These milestones could be *genesis_milestones* or issued after a particular interval. When a node received a milestone, it underwent rigorous validation. It checked the milestone’s signature using the coordinator’s public key to confirm its legitimacy. If the milestone passed this cryptographic scrutiny, the node integrated it into its local view of Tangle.

#### 3.5.4. Transaction Confirmation

Tangle’s consensus mechanism revolved around the confirmation status of a transaction. Transactions encapsulated within milestones and passing the network’s consensus rules were deemed ‘confirmed’, ensuring their permanent inclusion within Tangle and testing their validity.

#### 3.5.5. Double Spending Detection

To ensure the security of the network, nodes implemented specific mechanisms to identify and prevent double spending attempts. The *is_double_spent* function played a crucial role in this process. It operated by examining each incoming transaction against the node’s existing list of transactions. The function checked if the transaction attempted to use funds from an address that had already been spent or if the transaction ID matched any existing transaction in the node’s transaction list. Furthermore, the function extended its verification to the order of the transaction. It inspected not only the parents of the transaction but also their preceding transactions. By iterating through this chain of parent transactions, the function determined if the transaction’s ID appeared at any point in this order. If a match was found, indicating the reuse of the same transaction ID, the function identified a double spending attempt. This comprehensive check ensured both direct and ancestral transaction comparisons and effectively safeguarded the integrity of the ledger state, ensuring that each transaction was unique and funds were not illicitly reused.

#### 3.5.6. Pending Transaction Management

To effectively handle the missing transactions and the dynamic nature of the network, nodes maintained a queue of pending transactions. The *process_pending_transactions* method allowed nodes to continuously assess and process these transactions. Transactions were routinely checked, and those successfully processed were removed from the queue, ensuring that the system remained responsive and efficient. In addition, nodes were equipped with a method, *request_parent_transaction*, to retrieve missing parent transactions, essential in maintaining Tangle’s continuity. If a node encountered a transaction with a missing parent, it requested the missing transaction from its sender or peers. The corresponding method, *provide_missing_transaction*, enabled a node to locate a requested transaction in its list of transactions and forward it to the requester, ensuring the network’s integrity and flow of information.

## 4. Tangle Evaluation Results and Analysis

This section presents the outcomes of the simulations conducted on the specified components, emphasizing the significance of Poisson processes in the transaction dynamics, the analysis of random walks, node convergence, and the development of accumulative weights over time. These findings demonstrate the effectiveness of the simulator in analyzing the Tangle protocol, which was the primary protocol evaluated using our proposed simulator.

### 4.1. Network Analysis of Poisson Processes in Transaction Dynamics

In a decentralized network based on a DAG, nodes independently create and process transactions, primarily influenced by Poisson processes [14]. It is important to note that this analysis underscores the significance of discrete event simulators in such environments. While the studies referenced in Section 2 contribute to a collective understanding, they typically presume the network’s adherence to a Poisson distribution without empirical analysis. Our proposed simulator, however, allows for a meticulous, quantitative examination of this distribution, moving beyond mere assumptions to a more definitive analysis. For a given node *i*, the transaction generation rate can be denoted as $\lambda_{i}$. This leads to each node potentially generating transactions at a rate of λ transactions per second. Mathematically, the number of transactions $X_{i}$ generated by node *i* in time *t* conforms to $X_{i}∼\mathrm{Poisson}\left(\lambda_{i}t\right)$. Summatively, the aggregate number of transactions by all nodes in the network during time *t* is $X=\sum_{i=1}^{N}X_{i}$. This summation upholds the Poisson principle, making *X* Poisson-distributed with the parameter $\lambda =\sum_{i=1}^{N}\lambda_{i}$. The reception dynamics encompass network delays and peer-to-peer structures, yielding the effective reception rate at node *j* as

(3) $$\lambda_{j_{effective}}=\sum_{i=1,i\neq j}^{N}\lambda_{i}\times P\left(i\to j\right).$$

where P(i→j) signifies the likelihood of a transaction from node *i* reaching node *j*. With $Y_{j}$ denoting the transaction counts for node *j*, it is described as $Y_{j}∼\mathrm{Poisson}\left(\lambda_{j_{effective}}t\right)$. Network delay complexities might warrant a representation like

(4) $$\lambda_{j_{delayed}}=\lambda_{j_{effective}}\times \left(1-D\left(j\right)\right).$$

with D(j) being the mean delay for node *j*. The simulation approach relies on progressive state updates across nodes and involves iterating over each node to refresh the transaction state, symbolized by

(5) S(t+1)=f(S(t),λ,N).

**Figure 2:**
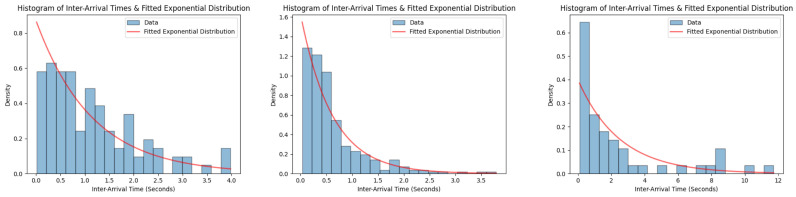
Inter-arrival times for different nodes over a specified simulation interval, demonstrating adherence to the Poisson distribution.

After the simulation, the mean transaction count per node can be computed as $\mu =\frac{1}{N}\sum_{j=1}^{N}Y_{j}$ and the observed count compared against a standard Poisson distribution using

(6) $$P\left(Y_{j}=k\right)=\frac{\mu^{k}e^{-\mu }}{k!}.$$

for k=0,1,2,... up to the maximum observed transaction count. This provides a comprehensive picture of the simulation approach. It not only models the dynamic behavior of the network but also statistically evaluates the observed results against the expected Poisson distribution and underscores its utility in understanding decentralized transaction dynamics. Figure 2 illustrates this comparison, showcasing histograms of inter-arrival times against fitted exponential distributions for three different nodes. While the results may not align perfectly with a pure Poisson distribution, they closely resemble a thinned version of the Poisson distribution, as described by Equation (6).

The parameter settings utilized in this analysis are summarized in Table 2. The interplay between these parameters and their influence on the network dynamics offer a richer insight into the decentralized transaction processes. Specifically, the range of delay values was chosen based on empirical evidence from IoT systems. According to [28], the delays in such systems predominantly range from 0.20 ms to <50 ms under typical circumstances. This analysis also accounted for extreme scenarios, pushing the upper limit to 90 ms to ensure a thorough evaluation of the system under potential edge conditions. Such granularity in the parameter choices ensured that the simulation captured a wide spectrum of real-world scenarios, enhancing the robustness and validity of our findings. In prior studies [29], λ has been traditionally considered as an upper bound, with values spanning between 100 and 10,000 transactions per unit of time, thereby deriving the transactions per node based on this aggregate upper limit. This methodology predominantly emphasizes a more holistic and macroscopic analysis. Contrarily, our implementation pivots toward a granular and discrete-level examination of individual entities within the network. Consequently, we tailored our analytical framework to scrutinize λ on an individual node level, with the transactions per second being categorized into two distinct ranges: 0.001–0.01 and 0.01–0.1. This nuanced approach offers a more microscopic perspective, shedding light on the intricate dynamics at play on a per-node basis.

### 4.2. Dynamics and Efficiency of the Random Walk in IOTA’s Tangle

The second aspect of our analysis using the proposed simulator focused on the random walk strategy within the Tangle protocol. It is important to clarify that the Tangle version examined is not the latest iteration released by IOTA [4]. The primary objective of this study is to establish the foundational elements of the simulator, which are designed to be adaptable to any DAG-based system. While the simulator can be extended to newer protocol versions, our choice to analyze this particular version is strategic. The aim is not merely to scrutinize the current Tangle iteration but to explore the fundamental factors that led IOTA to transition from this version to newer ones. We focus on the ‘what’ and ‘why’ of these changes, rather than the ‘how’, as the latter extends beyond the scope of this study. With these considerations in mind, we present an in-depth analysis of the random walk strategy employed in the Tangle protocol.

**Figure 3:**
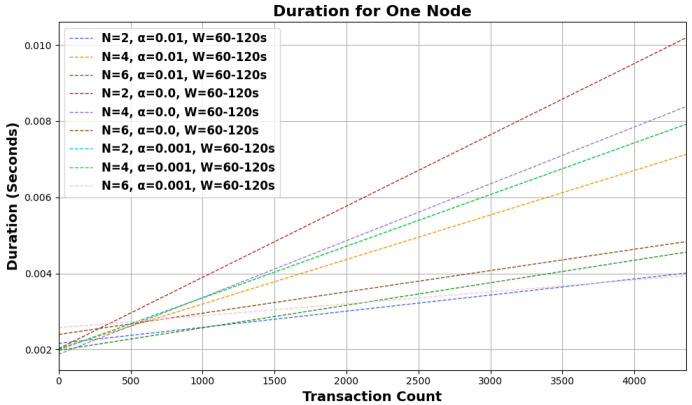
Random walk behavioral analysis across configurations for *W* = 60–120 s interval.

**Figure 4:**
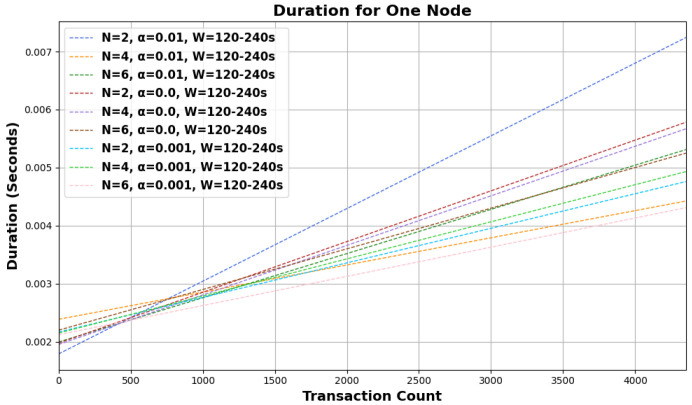
Random walk behavioral analysis across configurations for *W* = 120–240 s interval.

Based on the provided graphical representations in Figure 3 and Figure 4, the behavior of the random walk mechanism over different configurations and intervals can be observed. The graphs illustrate the ‘duration for one node’ concerning the ‘transaction count’ under various settings of *N*, α, and *W*.

Across both graphs, as the number of transactions increases, the duration for one node also rises linearly, suggesting a directly proportional relationship between the two. Furthermore, as *N*, the number of walkers, increases (from 2 to 6), the duration required for a single node also rises. This might be indicative of the overhead introduced by managing more walkers, even though more walkers would ideally mean a faster consensus. The variations in α, the bias parameter, showcase different trends in duration. A higher α seems to lead to a faster duration, especially evident for larger transaction counts. This implies that a higher degree of bias expedites the random walk process by driving it toward transactions with higher cumulative weights more quickly. Comparing the graphs, it can be noticed that the duration for one node is generally lower for intervals of *W* = 120–240 s than for *W* = 60–120 s under similar conditions, suggesting that shorter intervals lead to a quicker random walk process.

The influence of the number of walkers becomes more prominent as the bias parameter α decreases. In the graph with *W* = 60–120 s, the duration difference between N=2 and N=6 is more discernible for α=0.0 compared to α=0.01. With the longer interval of *W* = 120–240 s, the increase in duration for one node is more gradual across the transaction count, indicating that the system may have better efficiency or a smaller overhead over this extended timeframe. As outlined in Section 3, the asynchronous nature of dispatching walkers and determining the walk type likely contributes to the linear increase in duration. As more transactions are initiated, more walkers are dispatched simultaneously, leading to a consistent rise in the time taken to select tips.

The random walk mechanism, integral to Tangle’s operation, exhibits predictable behavior across varied configurations. While the linear relationship between the transaction count and duration underscores its scalability, the influences of *N*, α, and *W* highlight the intricacies of its operation. By understanding these nuances, optimizations can be made to further enhance the efficiency and responsiveness of the system in real-world applications. The foundational challenges highlighted in this study, through the use of the proposed simulator, offer a clear rationale for and understanding of the shift to the newer version of the Tangle protocol.

### 4.3. Quantitative Assessment of Node Convergence

In heterogeneous DAG networks, comprehending the dynamics of node convergence and synchronization and attaining a consistent state across the network are crucial in maintaining optimal network performance. This section clarifies the methodologies utilized in our proposed simulator to assess this synchronization. The focus is particularly on two key aspects: ‘tips’, which are the latest transactions that are yet to be approved, and ‘all_transactions’, representing the sum of all transactions received and generated by a node.

A critical initial step in this process was to clearly define the parameters for convergence. Determining whether nodes have reached convergence involves evaluating whether they have attained synchronization and a consistent state in terms of their transactional data. This was essential in ensuring that all nodes in the network possessed a coherent view of the ledger.

To quantitatively measure this synchronization and convergence, we introduced the pairwise overlap and convergence metric. This metric was designed to quantify the extent of overlap in the ‘tips’ and the convergence of ‘all_transactions’ across every pair of nodes within the network. The degree of overlap and convergence was expressed through specific equations, where each cell in the matrix, denoted as (i,j), represents the degree of overlap for ’tips’ and convergence for ’all_transactions’ between node *i* and node *j*. The formulas are given by

(7) TipsOverlap(i,j)=|TipsofNodei∩TipsofNodej|

(8) $$\begin{array}{cc} \mathrm{NodeConvergence}(i,j) & =\frac{\mathrm{Overlap}(i,j)}{\mathrm{TotalTransactions}}\\ \mathrm{Overlap}(i,j) & \text{:}=|B_{i}\cap B_{j}|\\ B_{i} & \text{:}=\mathrm{AllTransactions}\;\mathrm{of}\;\mathrm{Node}\;i\\ B_{j} & \text{:}=\mathrm{AllTransactions}\;\mathrm{of}\;\mathrm{Node}\;j \end{array}$$

To evaluate the average overlap and convergence, we calculated the mean overlap and convergence that each node had with the rest of the network. This provides insights into the mean ‘tips’ overlap and ‘all_transactions’ convergence for individual nodes with their peers:

(9) $$\mathrm{AvgTipsOverlap}\left(Node_{i}\right)=\frac{\sum \mathrm{Overlap}(i,j)}{N},\;\forall j\neq i$$

(10) $$\mathrm{AvgNodeConvergence}\left(Node_{i}\right)=\frac{\sum \mathrm{Convergence}(i,j)}{N},\;\forall j\neq i$$

For a normalized measure of overlap and convergence, we used the Jaccard Similarity, which evaluated the shared elements against the total unique elements between node pairs. This provided a more standardized approach to understanding overlap and convergence:

(11) $$\mathrm{JaccardTipsOverlap}\left(i,j\right)=\frac{\left|\mathrm{Tips}\;\mathrm{of}\;\mathrm{Node}\;i\cap \mathrm{Tips}\;\mathrm{of}\;\mathrm{Node}\;j\right|}{\left|\mathrm{Tips}\;\mathrm{of}\;\mathrm{Node}\;i\cup \mathrm{Tips}\;\mathrm{of}\;\mathrm{Node}\;j\right|}$$

(12) $$\begin{array}{cc} \mathrm{JaccardNodeConvergence}(i,j) & =\frac{\mathrm{Overlap}(i,j)}{\mathrm{Union}(i,j)}\\ \mathrm{where}\\ \mathrm{Overlap}(i,j) & =\;|A_{i}\cap A_{j}|\\ \mathrm{Union}(i,j) & =\;|A_{i}\cup A_{j}|\\ A_{i} & =\mathrm{AllTransactions}\;\mathrm{of}\;\mathrm{Node}\;i\\ A_{j} & =\mathrm{AllTransactions}\;\mathrm{of}\;\mathrm{Node}\;j \end{array}$$

Utilizing these methodological approaches in the proposed simulator provides a comprehensive understanding of node overlaps and convergence, essential in evaluating inter-node relationships in a DAG network. The precise application of these formulas is critical for accurate and standardized analysis, forming a fundamental aspect of any study in decentralized networks.

Table 3 presents an in-depth analysis of the convergence tendencies within a DAG, focusing on nodes’ behavior. The heterogeneous nature of DAGs poses a significant challenge in achieving convergence, as each node might have a distinct view of the network. Key parameters for this study included a gossip factor of 0.7, a probability for peers at 0.4, varying λ values from 0.01 to 0.1, and a total simulation duration of 3600 s (1 h). The results indicated that the ‘average overlap’ and ‘Jaccard similarity’ for all transactions generally were around 23% and 21%, respectively. While this uniformity seems promising, it raises concerns in the context of heterogeneous DAGs, questioning the network’s ability to reach a fully converged state. This finding highlights the inherent challenges in achieving a synchronized perspective on the DAG among nodes, exacerbated by the natural tendency of DAGs to support diverse node viewpoints.

In addressing these convergence challenges, IOTA’s implementation of the coordinator milestone is noteworthy. This mechanism serves as a reference point for nodes, aiding in harmonizing their views of the network, as discussed in [30]. Moreover, the new version of IOTA, which does not rely on milestones and coordinators [4], uses voting and a validator committee. However, this structure leads to thought-provoking questions: if convergence and a consistent network state are achieved through overarching mechanisms like the coordinator or validator committees, why is there a need for each node to independently engage in tip selection and perform PoW? Additionally, why is it essential for each node to maintain its own version of the DAG, especially in a network where a synchronized state is the objective?

### 4.4. Accumulative Weight Growth

**Figure 5:**
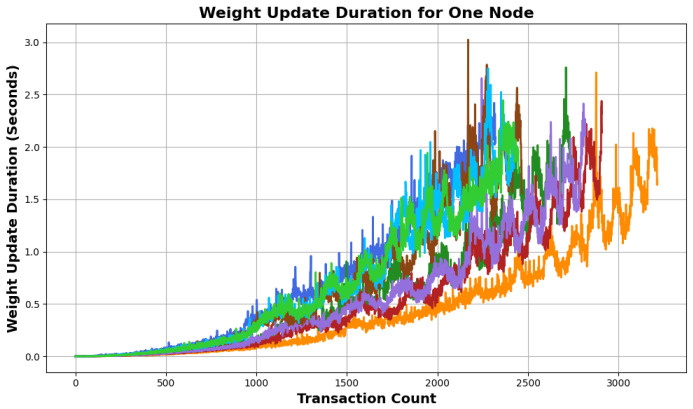
Weight update duration vs. transaction count: an analysis of computational overhead in accumulative weight calculations as a function of increasing transaction count.

The final metric that we tested using our proposed simulator was the accumulative weight, a critical aspect of the Tangle protocol. As Tangle evolves, efficient weight calculations become increasingly important. An evident observation from Figure 5 is the exponentially increasing duration of weight updates with the increasing size of Tangle. This progression indicates that as more transactions are added, the computational time to update the weights correspondingly increases. The colors in the figure map to parameters *W*, α, and *N* from Figure 3, highlighting that the weight calculation times are influenced by these configurations. Each node updates the weights locally, tied directly to the incoming tips, or λ. This underscores the direct proportionality of the weight computation time to λ. The increasing duration signifies the computational challenges. Therefore, future iterations and optimizations of Tangle might require strategies to streamline this process and ensure rapid weight updates despite an escalating number of transactions.

## 5. Discussion and Future Work

This section analyzes the complexities of updating weights and consensus in IOTA’s Tangle, focusing on the implications of the queue depth, memory needs, and network latency. We discuss the challenges of decentralizing consensus, including the system overhead, node heterogeneity, and validator diversity. Additionally, we highlight the study limitations and suggest future research directions, such as investigating security threats, exploring alternative tip selection algorithms, and considering a multi-validator approach.

### 5.1. Weight Update without Depth Limitation

Based on Section 3.2.3 and Section 4.4, the accumulative weight plays an important role in the confirmation of a transaction. As outlined in the IOTA white paper, the accumulative weight is designed to incrementally increase as new transactions are added to the DAG. To further investigate the impact of accumulative weight increments, we consider *T* as the total number of transactions in Tangle up to a given time and *t* to indicate that a new transaction is added. Each transaction confirms two previous transactions. If we assume the worst-case scenario without depth limitation, the weight of each transaction would need updating till genesis. Thus, for a transaction *t*, the total number of weight updates, $U_{t}$, would be

(13) $$U_{t}=2\times \sum_{i=1}^{T}\lambda_{i}$$

where $\lambda_{i}$ represents the weight contribution of the ith transaction to *t*.

#### 5.1.1. Time Complexity with Queue Depth

Transactions are not processed immediately but are placed in a queue. Let us denote the queue depth (the number of transactions waiting to be processed) as *Q*.

The time to update the weights, assuming an average queue processing time of δ per transaction, would be

(14) $$\tau \left(t\right)=k\times U_{t}+Q\times \delta$$

where *k* is the average time taken to update a single transaction’s weight.

Since *Q* can vary dynamically based on the network load and other factors, the time complexity becomes a function of both *T* and *Q*:

(15) $$\tau \left(t\right)=k\times \left(2\times \sum_{i=1}^{T}\lambda_{i}\right)+Q\times \delta$$

This implies higher variability in transaction confirmation times as Tangle grows.

#### 5.1.2. Memory Requirements with Shard Overhead

Considering the presumption that each node computes the accumulative weight locally, the node must maintain a complete copy of the DAG up to the genesis block, thereby raising potential memory concerns. Nonetheless, several techniques, including sharding [31,32], have been proposed to address these challenges. With the prospect of sharding in distributed ledgers, let us consider the overhead introduced by sharding. *s* is the base size (in memory) of a single transaction and ω is the overhead factor for each shard.

If we assume *S* shards,

(16) M(T)=s×T+ω×s×S

This indicates that, while sharding may distribute the load, it introduces an overhead that could affect the memory requirements.

#### 5.1.3. Latency Incorporating Network Effects

Network latency plays a pivotal role in the time taken to calculate and update the accumulative weights of transactions. Let us define ϵ as the average network latency and ρ as the proportion of nodes that need to update their local Tangle copy for consensus, specifically to incorporate the changes in accumulative weight.

Given the importance of accumulative weight calculations, the latency *L* due to network effects for a transaction *t* can be represented as

(17) L(t)=α×τ(t)+β+ρ×ϵ

where α is a proportionality constant, denoting the time taken for the internal computation associated with accumulative weight calculations. β represents the time required for I/O operations, specifically for the reading and updating of the accumulative weights in the system.

The accumulative weight mechanism, while fundamental in Tangle’s design, introduces complexities in time, memory, and network latency. Tangle’s scalability, while promising, encounters challenges both in time complexity and memory requirements as the system grows. Factors such as queue depths and sharding introduce variability that needs to be managed to maintain consistent performance. Network effects, especially in globally distributed systems, add another layer of complexity to the overall performance of the system. Proper protocols and optimizations are essential to ensure that Tangle remains viable for large-scale applications.

### 5.2. Challenges and Implications of Consensus in DAG-Based Systems

As Section 3.2.5 discusses, the role of the coordinator/validators in Tangle is pivotal. It serves as an interim solution to safeguard against double spending and potential vulnerabilities and ensure a single confirmed state of the ledger. Despite the coordinator’s utility in ensuring the network’s security, the overarching vision for IOTA is a coordinator-free environment, such as in ‘The Coordicide’ [33] and ‘Tangle 2.0 Leaderless’ [4]. This aspiration is not merely aimed at embracing decentralization in its purest form, but also at optimizing the system’s efficiency, eliminating potential bottlenecks, and ensuring a homogenized validation process. While several approaches integrating voting and permitted validator committees exist and warrant deeper investigation, this study’s focus remains on the coordinator. The appeal of DAG-based systems lies in their scalability and potential for genuine decentralization. However, the introduction of coordinators or permitted validator committees introduces significant complexities, posing challenges to their seamless implementation.

#### 5.2.1. Potential System Overhead

If the coordinator/validator is seen as the final authority, the cumulative security efforts at each node, as previously described (Section 5.1), could be perceived as redundant. The original intent behind local weight calculations and validations was to democratize security, making every participant equally responsible for the network’s security. If the coordinator/validator’s decisions ultimately supersede all others, then the intrinsic value of these decentralized efforts might be negated, leading to system inefficiencies.

#### 5.2.2. Heterogeneity in the DAG

By design, Tangle does not impose strict uniformity across its nodes. As the node convergence Section 4.3 suggests, each node in the IOTA network possesses a distinct local view of Tangle [34]. While this design promotes decentralization and scalability, it presents challenges for a centralized entity like the coordinator.

#### 5.2.3. Multiplicity of Validator and Consistency Challenges

The idea of employing multiple validators could potentially exacerbate the issue of network consistency. A solitary coordinator, while presenting a single point of failure or control, ensures that milestones are consistently recognized across the network. Introducing more validators, given the intrinsic heterogeneity of Tangle, means that each validator would likely have a disparate view of Tangle due to the asynchronous nature of transactions and validations. As a consequence, different validators might generate varying milestones or voting, possibly referencing diverse sets of transactions. This can lead to inconsistencies regarding which transactions are deemed ‘confirmed’, creating potential disparities in the perceived state of the ledger across nodes.

### 5.3. Future Work and Limitations

While the current study robustly covers the foundational protocols of Tangle, there remains substantial scope to extend this work. One primary avenue is the incorporation of malicious node activities, enabling a deeper exploration of potential vulnerabilities, the analysis of newer versions of Tangle, and the system’s resilience against various attacks. Additionally, given our intention to establish a benchmark for discrete event simulation, it would be pertinent to integrate alternative tip selection algorithms. This would allow for a comprehensive understanding of DAG-based distributed ledger dynamics, transcending the confines of IOTA and providing insights into a broader spectrum of distributed ledgers. Furthermore, in our current implementation, we primarily employ a single coordinator for Tangle’s operation. Looking ahead, we anticipate transitioning to a model with multiple validators. This transition will inherently introduce added complexities. Moreover, the potential for network latency becomes more pronounced with multiple active validators, which could impact Tangle’s optimal performance. Future studies will also focus on conducting a comprehensive quantitative analysis of the cumulative weight growth and network effects, employing sophisticated mathematical and computational models to substantiate the observed phenomena. Navigating these complexities will be essential, but, by addressing them, we aim to further our understanding and optimization of DAG-based distributed ledger systems.

## 6. Conclusions

Through our detailed evaluation of the IOTA Tangle protocol, guided by its white paper, we delved into the complexities of DAG-based distributed ledgers. Utilizing a purpose-built discrete-event-based simulator, our analysis revealed that subtle intricacies within DAG architectures can be uncovered.

This comprehensive analysis encompasses the observation of the scalability of the random walk mechanism, its sensitivity to the walker count *N*, the impact of bias α on responsiveness, and the efficiency gains achieved with shorter time intervals *W*. These findings illuminate the foundational challenges and optimization opportunities within the protocol, providing a solid rationale for its evolution. Additionally, we have studied the autonomous node operations, guided by Poisson processes. This analysis underscores the crucial role of discrete event simulators in understanding the dynamics of such decentralized environments. Unlike previous studies that often assume a network’s adherence to a Poisson distribution without empirical validation, our proposed simulator enables a quantitative examination of this distribution. We have demonstrated how individual node transaction generation rates $\lambda_{i}$ contribute to the aggregate transaction rate λ for the entire network, offering a more granular perspective. The comparisons suggest that while the results may not perfectly align with a pure Poisson distribution, they closely resemble a thinned version of it.

These mechanisms, while innovative in transaction confirmations, present challenges, especially as Tangle grows, introducing higher time complexity and memory demands. Potential solutions such as sharding, though promising, introduce their own overheads, which might impact system latency. As Tangle evolves, addressing these issues becomes crucial in ensuring seamless and consistent performance.

Moreover, our evaluation of the coordinator/validator highlights its indispensable role while underscoring potential challenges inherent to its function. Its protective role is undeniable, but its centralized nature poses a dichotomy in a decentralized system.

Looking ahead, we see numerous avenues for deeper exploration. Understanding malicious node behaviors, assessing system vulnerabilities, and weighing the benefits and disadvantages of different tip selection algorithms will be crucial.

In summary, our research underscores that DAG-based distributed ledgers hold significant potential due to their scalability, especially for IoT applications. However, addressing the challenges outlined in this study is crucial for the widespread adoption of this technology. Our study serves as a directional guide, elucidating the complexities of DAG-based systems and indicating potential possibilities for subsequent research and development. The extensive nature of our proposed simulator, with its comprehensive analysis capabilities, is a valuable asset in further investigating these challenges. Its application can provide deeper insights into the dynamics of DAG networks, aiding in the optimization of these systems for broader, practical deployment.

## Figures and Tables

**Table 1:** Comparison of DAG-based simulators in related work.

Work	Year	Focus and Key Features	Model	DE
Tangle Simulator	2018	Tangle, stability of tip counts against various tip selection methods with limited configuration options.	Continuous-time	×
CIDDS	2018	Extension of Tangle Simulator with enhanced configuration options and a larger number of transactions, investigates throughput and networking layer.	Continuous-time	×
BlockSim	2019	Linear blockchain, aims to mimic and analyze linear blockchains.	Software tool	×
DagSim	2019	Tangle, asynchronous for DAG-based cryptocurrencies by introducing honest and semi-honest agents.	Continuous-time	×
Ext. Blocksim	2021/22	Linear blockchains, extension of Blocksim for hybrid blockchains and segwit extension.	Software tool	×
TangleSim	2023	Tangle 2.0, explores attack scenarios under diverse network topologies, packet losses, and a gossip protocol.	Agent-based	×
DAGSword	2023	Phantom miner and block rewards, built on existing linear Bitcoin simulator with focus on miners, block rewards, and mempool optimization.	Discrete-event	✓
MAIOTSim	2024	IOTA, providing the security verification of consensus under double spending attack scenarios.	Multi-agent	×
Our Work	2024	Generic DAG, proposed generic implementation of DAG data structure can easily be extended to multiple DAG-based distributed ledgers; analyzes two tip selection methods, accumulative weight and Poisson distribution.	Agent-based and discrete-event	✓

**Table 2:** Parameter settings for Poisson distribution analysis.

Parameter	Values
Lambda	0.001–0.01, 0.01–0.1
Number of Nodes	10, 100, 500, 1000
Delay (ms)	0.20–0.90, 0.90–4.0,4.0–50.0, 50.0–90.0
Routing Protocol	Gossip Protocol

**Table 3:** Observations of overlap and Jaccard similarity over time.

Time(s)	Tips—Average Overlap	Tips—Jaccard Similarity	All Transactions—Average Overlap	All Transactions—Jaccard Similarity
252	15.5%	14.4%	23.44%	21.77%
1217	15.2%	14.2%	23.27%	21.60%
1586	15.8%	14.7%	23.31%	21.65%
2072	15.5%	14.4%	23.29%	21.63%
2437	15.6%	14.5%	23.29%	21.63%
2496	15.5%	14.4%	23.28%	21.62%
2782	15.5%	14.4%	23.28%	21.62%
3313	15.7%	14.6%	23.30%	21.64%
3369	15.5%	14.4%	23.30%	21.64%
3542	15.3%	14.2%	23.30%	21.64%

## Data Availability

The complete implementation and implementation specification document can be accessed at the GitHub Repository (https://github.com/Misbah-khan786/DAGNetSim (accessed on 14 January 2024)).

## Abbreviations

The following abbreviations are used in this manuscript:

DAG	Direct Acyclic Graph
IoT	Internet of Things
DE	Discrete Event
AW	Accumulative Weight
OW	Own Weight
BRW	Biased Random Walk
URW	Unbiased Random Walk
MCMC	Markov Chain Monte Carlo
RW	Random Walker
PoW	Proof of Work

## References

[1] Popov S. (2018). The tangle. White Paper.

[2] LeMahieu C. (2018). Nano: A Feeless Distributed Cryptocurrency Network. Nano.

[3] Schueffel P. (2017). Alternative distributed ledger technologies Blockchain vs. Tangle vs. Hashgraph-A high-level overview and comparison. Tangle vs. Hashgraph-A High-Level Overview and Comparison. https://papers.ssrn.com/sol3/papers.cfm?abstract_id=3144241.

[4] Müller S., Penzkofer A., Polyanskii N., Theis J., Sanders W., Moog H. (2022). Tangle 2.0 leaderless nakamoto consensus on the heaviest dag. IEEE Access.

[5] Sompolinsky Y., Wyborski S., Zohar A. PHANTOM GHOSTDAG: A scalable generalization of Nakamoto consensus: September 2, 2021. Proceedings of the 3rd ACM Conference on Advances in Financial Technologies.

[6] Zander M., Waite T., Harz D. (2019). DAGsim: Simulation of DAG-based distributed ledger protocols. ACM Sigmetrics Perform. Eval. Rev..

[7] Lin B.Y., Dziubałtowska D., Macek P., Penzkofer A., Müller S. TangleSim: An Agent-based, Modular Simulator for DAG-based Distributed Ledger Technologies. Proceedings of the 2023 IEEE International Conference on Blockchain and Cryptocurrency (ICBC).

[8] Perešíni M., Hladkỳ T., Malinka K., Homoliak I. (2023). DAG-Sword: A Simulator of Large-Scale Network Topologies for DAG-Oriented Proof-of-Work Blockchains. arXiv.

[9] Andresen G. (2015). Bitcoin Mining Simulator Simulator. https://github.com/gavinandresen/bitcoin_miningsim.

[10] Ma X., Wu H., Xu D., Wolter K. CBlockSim: A Modular High-Performance Blockchain Simulator. Proceedings of the 2022 IEEE International Conference on Blockchain and Cryptocurrency (ICBC).

[11] Alharby M., Van Moorsel A. (2019). Blocksim: A simulation framework for blockchain systems. ACM Sigmetrics Perform. Eval. Rev..

[12] Polge J., Ghatpande S., Kubler S., Robert J., Le Traon Y. (2021). Blockperf: A hybrid blockchain emulator/simulator framework. IEEE Access.

[13] Basile M., Nardini G., Perazzo P., Dini G. SegWit extension and improvement of the BlockSim Bitcoin simulator. Proceedings of the 2022 IEEE International Conference on Blockchain (Blockchain).

[14] Fan C., Ghaemi S., Khazaei H., Chen Y., Musilek P. (2021). Performance analysis of the IOTA DAG-based distributed ledger. ACM Trans. Model. Perform. Eval. Comput. Syst..

[15] Nguyen M.N. (2018). Tanglesimulator. https://github.com/minh-nghia/TangleSimulator.

[16] Lathif M.R.A., Nasirifard P., Jacobsen H.A. Cidds: A configurable and distributed dag-based distributed ledger simulation framework. Proceedings of the 19th International Middleware Conference (Posters).

[17] Li S., Xu H., Li Q., Han Q. (2024). Simulation study on the security of consensus algorithms in DAG-based distributed ledger. Front. Comput. Sci..

[18] Guo F., Xiao X., Hecker A., Dustdar S. Characterizing IOTA tangle with empirical data. Proceedings of the GLOBECOM 2020-2020 IEEE Global Communications Conference.

[19] Guo F., Xiao X., Hecker A., Dustdar S. (2022). A Theoretical Model Characterizing Tangle Evolution in IOTA Blockchain Network. IEEE Internet Things J..

[20] PS Foundation Asyncio—Asynchronous I/O. 2001–2024. https://docs.python.org/3/library/asyncio.html.

[21] PS Foundation Python Threading. 2001–2024. https://docs.python.org/3/library/threading.html#module-threading.

[22] Schönbrodt F.D., Humberg S. (2023). RSA: An R Package for Response Surface Analysis.

[23] Harris C.R., Millman K.J., van der Walt S.J., Gommers R., Virtanen P., Cournapeau D., Wieser E., Taylor J., Berg S., Smith N.J. (2020). Array programming with NumPy. Nature.

[24] Hagberg A.A., Schult D.A., Swart P.J. Exploring Network Structure, Dynamics, and Function using NetworkX. Proceedings of the 7th Python in Science Conference.

[25] Hunter J.D. (2007). Matplotlib: A 2D graphics environment. Comput. Sci. Eng..

[26] Research A. (2008). Graphviz-Graph Visualization Software.

[27] Cohen B. Incentives build robustness in BitTorrent. Proceedings of the Workshop on Economics of Peer-to-Peer systems.

[28] Alaslani M., Nawab F., Shihada B. (2019). Blockchain in IoT systems: End-to-end delay evaluation. IEEE Internet Things J..

[29] Kusmierz B., Sanders W., Penzkofer A., Capossele A., Gal A. Properties of the tangle for uniform random and random walk tip selection. Proceedings of the 2019 IEEE International Conference on Blockchain (Blockchain).

[30] Conti M., Kumar G., Nerurkar P., Saha R., Vigneri L. (2022). A survey on security challenges and solutions in the IOTA. J. Netw. Comput. Appl..

[31] Naresh V.S., Allavarpu V.D., Reddi S. (2022). Blockchain IOTA Sharding-Based Scalable Secure Group Communication in Large VANETs. IEEE Internet Things J..

[32] Sealey N., Aijaz A., Holden B. IOTA Tangle 2.0: Toward a Scalable, Decentralized, Smart, and Autonomous IoT Ecosystem. Proceedings of the 2022 International Conference on Smart Applications, Communications and Networking (SmartNets).

[33] Popov S., Moog H., Camargo D., Capossele A., Dimitrov V., Gal A., Greve A., Kusmierz B., Mueller S., Penzkofer A. (2020). The Coordicide. http://files.iota.org/papers/20200120_Coordicide_WP.pdf.

[34] Müller S., Amigo I., Reiffers-Masson A., Ruano-Rincón S. (2023). Stability of local tip pool sizes. arXiv.

